# Kearns Sayre syndrome: a rare etiology of complete atrioventricular block in children (case report)

**DOI:** 10.11604/pamj.2021.40.154.24281

**Published:** 2021-11-15

**Authors:** Hanane Kharbouch, Badr Boussaadani, Ibtissam Fellat, Latifa Oukerraj, Nawal Doghmi, Mohamed Cherti

**Affiliations:** 1 Cardiovascular Diseases B Department, Ibn Sina Medical Hospital, Mohamed V University, Rabat, Morocco

**Keywords:** Kearns Sayre syndrome, atrioventricular block, peacemaker implantation, implantable automatic defibrillator, case report

## Abstract

Kearns Sayre syndrome is a rare mitochondrial abnormality first described in 1958, characterized by a triad associating progressive external ophthalmoplegia, ptosis, and pigmentary retinopathy with progressive alteration of cardiac conduction, which determines the vital prognosis of this entity. Here we report the case of a 13-year-old child of consanguineous parents who consults for recurrent syncope. The clinical exam found bilateral ptosis with complete atrioventricular block on electrocardiogram. The ophthalmological exam found pigmentary retinopathy. The patient underwent successful implantation of a double chamber pacemaker within 24 hours of admission, with an uneventful postoperative course. This case report highlights the interest of systematically assessing cardiac complications in children with mitochondrial disease such as Kearns Sayre syndrome, especially since cardiac involvement is the major prognostic factor in this disease.

## Introduction

Kearns-Sayre Syndrome (KSS) is a rare mitochondrial abnormality, characterized by progressive onset of external ophthalmoplegia, progressive pigment degeneration of the retina, and progressive alteration of cardiac conduction which is the main prognostic factor [1]. This disease is associated with a high risk of sudden death following complete atrioventricular block. Here we report a rare case of Kearns Sayre Syndrome revealed by complete atrioventricular block.

## Patient and observation

**Patient information:** our case is about a 13-year-old boy, 2^nd^ of 4 children with 1^st^ degree consanguineous parents. The pregnancy and childbirth (vaginal delivery) were uneventful, with good weight and psychomotor development. The child presented since the age of 5 months´ bilateral progressive ptosis. Five months before consultation in our department, he presented a repetitive syncope and lipothymic discomfort, occurring as well at rest as in physical effort.

**Figure 1:**
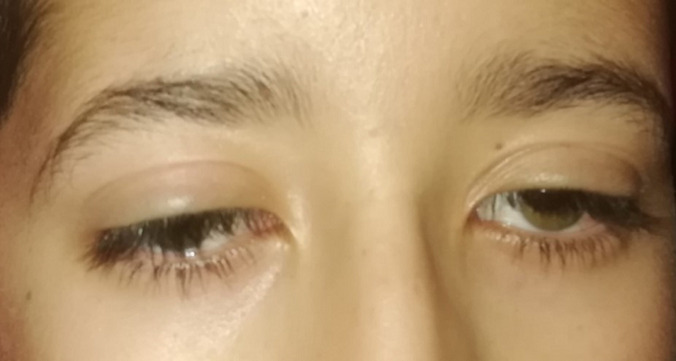
bilateral ptosis

**Figure 2:**
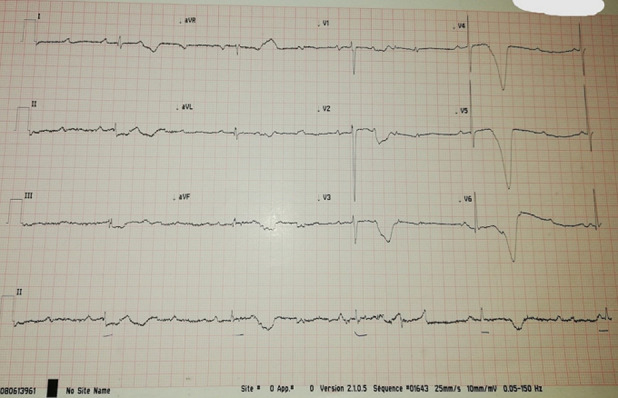
electrocardiogram of complete atrioventricular block

**Clinical findings:** the clinical assessment found a bilateral ptosis (Figure 1), normal cardiac auscultation, and normal neurological assessment. The Electrocardiogram (ECG) revealed a 3^rd^ -degree atrioventricular block with an average ventricular rate at 30 b/min (Figure 2).

**Figure 3:**
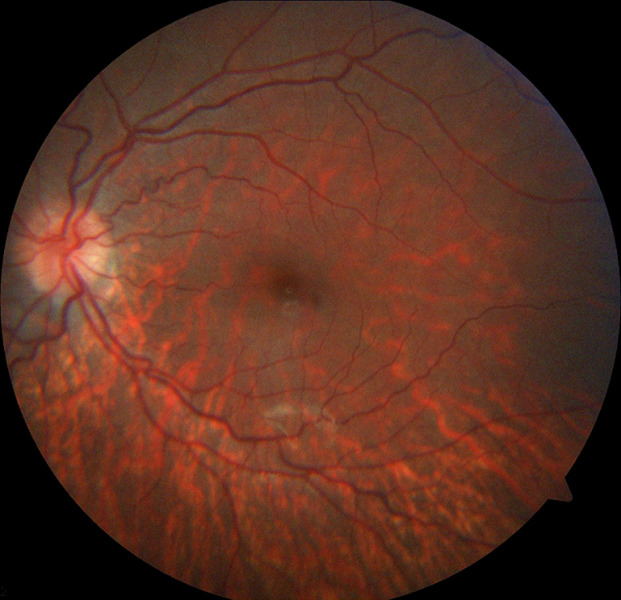
ophthalmological exam, pigmentary retinopathy

**Diagnostic assessment:** the echocardiography showed a dilated left ventricle with preserved function with no congenital malformations. The biological assessment is without abnormalities, including thyroid testing, hepatic balance, glycemia, lactic acid, renal function, and blood cell count. As part of the etiological assessment of the Atrioventricular Block (AVB) and in the presence of bilateral ptosis, an ophthalmological assessment was carried, showing pigmentary retinopathy (Figure 3). The KSS diagnosis was retained in our patient following clinical triad: bilateral ptosis, pigmentary retinopathy, cardiac conduction disorder occurring in childhood.

**Figure 4:**
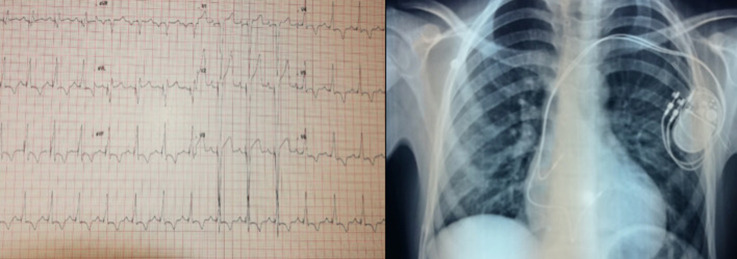
electrocardiogram and chest X-ray following pacemaker implantation, electro entrained rhythm

**Therapeutic intervention and follow up:** the patient underwent successful implantation of a double chamber pacemaker within 24 hours of admission, with an uneventful postoperative course (Figure 4). Clinical assessment at 3 months was without abnormalities.

## Discussion

KSS represents a progressive multi-systemic disorder related to mitochondrial abnormality, often occurring before the age of 20 years. This rare entity associates a triad made of external ophthalmoplegia, retinopathy, and atrioventricular block which conditions the prognosis. Additional features can be associated including cerebellar ataxia, impaired cognitive disability, dementia, sensorineural hearing loss, ptosis, oropharyngeal and oesophageal dysfunction, exercise intolerance, muscle weakness, and endocrinopathy [2].

KSS corresponds to mitochondrial cytopathy related to the deletion of mitochondrial Deoxyribonucleic Acid (DNA) of striated muscles, but also of the myocardium, central or peripheral nervous system, skin, and the retinal pigment epithelium [3]. The prevalence of this disease is about 1 to 3 per 100 000 individuals [4]. Patients with KSS are 20% more likely to have syncope and sudden death than the general population. Cardiac involvement in KSS is variable, about 50% of patients develop conductive abnormalities leading to complete atrioventricular block or bradycardia-related polymorphic ventricular tachycardia [5] and may also include dilated cardiomyopathy requiring a ventricular assist device [6-10].

In our patient, KSS was revealed following recurrent syncope, which suggests the possibility of underlying intermittent atrioventricular conduction disease. The syncope in KSS should be carefully assessed because of the high possibility of conduction system involvement. Cardiac involvement is the main prognostic factor of KSS, with variable age of cardiac symptoms' onset. Ingrid *et al*. found an average age of 28 years, ranging from 9 to 47 years, and this was not correlated with the age of disease onset [1]. Therefore, it is clear that the severity of disease correlates well with the severity of cardiac involvement. However, the progression towards a high-grade AVB remains variable and unpredictable, it may be rapidly progressive with an evolution towards a 100% dependent pacemaker rhythm following few months [7], Polak *et al*. reported a delay of 10 months [8].

Few Cases of sudden death have recently been reported in KSS patients despite the implantation of a pacemaker highlighting the risk of developing a ventricular arrhythmia (polymorphic or monomorphic ventricular tachycardia, episodes of *torsades de pointes* in the setting of prolonged QTc) suggesting the interest of cardiac Magnetic Resonance Imaging (MRI) in assessing left ventricular fibrosis. In the absence of obvious ventricular arrhythmias requiring an implantable automatic defibrillator (IAD), cardiac MRI should be considered before implantation of a pacemaker. In the presence of myocardial scarring signs, an intra cardiac electrophysiological study could be useful in predicting the need of an IAD as opposed to a pacemaker [6, 1-9].

## Conclusion

Cardiac involvement in KSS is associated with a high risk of syncope and sudden cardiac death, hence the importance of assessing syncope in suspected KSS patients. This cardiac involvement is variable and requires appropriate management. Our patient underwent early successful pacemaker implantation. However, the IAD has not been implanted in the absence of clear recommendations for primary prevention, he will be the subject of further exploration and close follow-up.
